# PubChemLite Plus Collision Cross Section (CCS) Values for Enhanced Interpretation of Nontarget Environmental Data

**DOI:** 10.1021/acs.estlett.4c01003

**Published:** 2025-01-24

**Authors:** Anjana Elapavalore, Dylan H. Ross, Valentin Grouès, Dagny Aurich, Allison M. Krinsky, Sunghwan Kim, Paul A. Thiessen, Jian Zhang, James N. Dodds, Erin S. Baker, Evan E. Bolton, Libin Xu, Emma L. Schymanski

**Affiliations:** † Luxembourg Centre for Systems Biomedicine (LCSB), University of Luxembourg, 6 Avenue du Swing, 4367 Belvaux, Luxembourg; ‡ Department of Medicinal Chemistry, University of Washington, Seattle, Washington 98195, United States; § Current Address: Biological Sciences Division, Pacific Northwest National Laboratory, Richland, Washington 99352, United States; ∥ National Center for Biotechnology Information (NCBI), National Library of Medicine (NLM), National Institutes of Health (NIH), Bethesda, Maryland 20894, United States; ⊥ Department of Chemistry, University of North Carolina, Chapel Hill, North Carolina 27599, United States

**Keywords:** nontarget screening, identification, PubChemLite, exposomics, ion mobility, collision cross section, PubChem

## Abstract

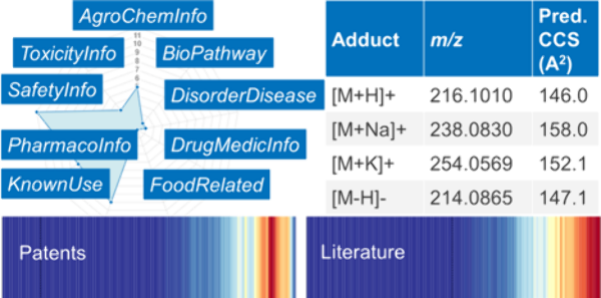

Finding relevant chemicals in the vast (known) chemical space is a major challenge for environmental and exposomics studies leveraging nontarget high resolution mass spectrometry (NT-HRMS) methods. Chemical databases now contain hundreds of millions of chemicals, yet many are not relevant. This article details an extensive collaborative, open science effort to provide a dynamic collection of chemicals for environmental, metabolomics, and exposomics research, along with supporting information about their relevance to assist researchers in the interpretation of candidate hits. The PubChemLite for Exposomics collection is compiled from ten annotation categories within PubChem, enhanced with patent, literature and annotation counts, predicted partition coefficient (logP) values, as well as predicted collision cross section (CCS) values using CCSbase. Monthly versions are archived on Zenodo under a CC-BY license, supporting reproducible research, and a new interface has been developed, including historical trends of patent and literature data, for researchers to browse the collection. This article details how PubChemLite can support researchers in environmental and exposomics studies, describes efforts to increase the availability of experimental CCS values, and explores known limitations and potential for future developments. The data and code behind these efforts are openly available. PubChemLite can be browsed at https://pubchemlite.lcsb.uni.lu.

## Introduction

Environmental and exposomics researchers are faced with the daunting task of determining which chemicals, among other factors, may be either potentially detrimental or beneficial in the context of human and environmental health. Nontarget screening (NTS) methods leveraging high resolution mass spectrometry (HRMS) approaches are now commonly used to explore complex samples due to high sensitivity and selectivity plus improved availability of HRMS instruments.^1,2^ Ion mobility spectrometry (IMS), which separates molecules based on their size and shape, is increasingly accessible, with the calculated collision cross section (CCS) values serving as an additional parameter to support identification in NTS.^3−5^ Nonetheless, the identification and - importantly - interpretation of features detected during NTS is still challenging, hindering the broader adoption of NTS.^1^ Identification in NTS primarily relies on mass spectral libraries, relatively small suspect lists and large chemical databases, as recently reviewed elsewhere.^1,2^ While the integration of IMS/CCS into workflows generally remains relatively poor, several methods to predict CCS values are now available to alleviate this situation, including quantum (*e.g.,* MobCal^6^ and ISiCLE^7^) and machine learning (ML) methods (*e.g.,* AllCCS,^8^ CCSbase,^9^ SigmaCCS,^10^ DeepCCS,^11^ and CCS Predictor 2.0^12^).

Compound databases commonly used in NTS include (numbers as of Dec. 2024) HMDB^13^ (220,945 metabolites), CompTox^14^ (1,218,248 chemicals), PubChem^15^ (119 million compounds), and ChemSpider^16^ (129 million structures). The Chemical Abstract Services (CAS) Registry^17^ (219 million chemicals) is licensed and thus unavailable to open workflows. Since ChemSpider introduced programmatic access limitations in 2018, PubChem has become the *de facto* standard large chemical database for open-science-based NTS methods. While PubChem, with >1000 sources, integrates the contents of many of the smaller openly available databases, PubChem also includes tens of millions of entries that are neither likely to be found in the environment, nor pertinent to the exposome. This hinders both the performance and efficiency of NTS. Additionally, many other potential sources for NTS identification efforts, such as the Global Chemical Inventory (350,000 chemicals)^18^ and various lists from European regulators contributing to the NORMAN Suspect List Exchange (NORMAN-SLE)^19^ include large proportions of chemicals that have very little supporting evidence about their existence and relevance, which makes interpretation of potential hits in NTS very challenging.

To mitigate these challenges, a subset of PubChem called PubChemLite was developed specifically to streamline NTS identification and interpretation.^20^ PubChemLite has been integrated into existing HR-MS workflows, such as patRoon^21^ and MetFrag.^22^ Although PubChemLite is familiar to many researchers already, the original 2021 article^20^ was primarily technical. This article explains PubChemLite for an environmental/exposomics audience, describes the integration of predicted CCS values in PubChemLite to support IMS, introduces the development of an open experimental CCS pipeline in PubChem to provide more experimental data for future CCS predictions, and presents a new web interface (https://pubchemlite.lcsb.uni.lu/) to browse the contents of PubChemLite.

## Methods and Materials

### Building PubChemLite

**Figure 1:**
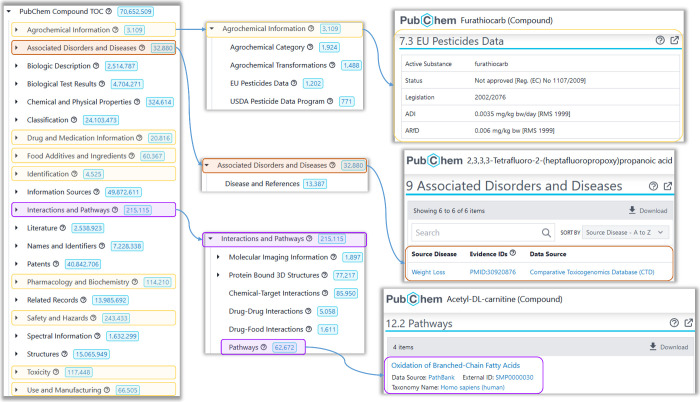
PubChemLite categories in the PubChem Table of Contents (TOC) (https://pubchem.ncbi.nlm.nih.gov/classification/#hid=72), selected subcategories, and associated annotation examples. Yellow shading denotes “environmental” categories (example CID 47759) (https://pubchem.ncbi.nlm.nih.gov/compound/47759#section=EU-Pesticides-Data), red the “exposomics” (example CID 114481) (https://pubchem.ncbi.nlm.nih.gov/compound/114481#section=Associated-Disorders-and-Diseases) and purple the “metabolomics” sections (example CID 1) (https://pubchem.ncbi.nlm.nih.gov/compound/1#section=Pathways). For high resolution live images, please click the embedded hyperlinks. Logo image from GitLab.^28^

The full technical details of PubChemLite are published elsewhere.^20^ Briefly, PubChemLite is derived from major categories relevant to environmental/exposomics applications appearing in the PubChem Table of Contents (TOC) (https://pubchem.ncbi.nlm.nih.gov/classification/#hid=72) pages.^23^ The ten categories currently used to compile PubChemLite (see Figure 1) are Agrochemical Information (AgroChemInfo), Associated Disorders and Diseases (DisorderDisease), Drug and Medication Information (DrugMedicInfo), Food Additives and Ingredients (FoodRelated), Identification (Identification), Interactions and Pathways—Pathways subset (BioPathway), Pharmacology and Biochemistry (PharmacoInfo), Safety and Hazards (SafetyInfo), Toxicity (ToxicityInfo), and Use and Manufacturing (KnownUse). These categories have remained consistent since the original publication, except for the “Biomolecular Interactions and Pathways” category, which was renamed by PubChem to “Interactions and Pathways” in 2022, then limited to the Pathways subset in May 2023 (see Results and Discussion). The input files (https://gitlab.com/uniluxembourg/lcsb/eci/pubchemlite-input)^24^ and code for the PubChemLite build system (https://gitlab.com/uniluxembourg/lcsb/eci/pclbuild)^25^ are available on the Environmental Cheminformatics (ECI) GitLab (https://gitlab.com/uniluxembourg/lcsb/eci/)^26^ repository (see Data Availability Statement).

Any compound with one or more of the selected annotation categories is included. The matching compounds (represented by PubChem Compound IDentifiers, CIDs) are aggregated by the first block of InChIKey into a primary entry and related CIDs, where the primary entry is the neutral or “parent” form. Entries such as mixtures and disconnected substances are excluded (see the original publication^20^ for details). Chemical information (SMILES, InChI, InChIKey, formula, mass), patent, and literature (PubMed) counts plus predicted XlogP values are retrieved in bulk. The chemical identifiers, mass, and XlogP values correspond to the “parent” (primary entry), while the annotation, patent, and literature counts are aggregated across all related CIDs. Importantly, the presence of an entry in PubChemLite means that at least one of these annotation categories is available for each CID, with the resulting information publicly available on PubChem to help interpret the relevance of the candidate, see Figure 1. PubChemLite is built and evaluated each week, with one public release per month.^27^

### Adding Predicted CCS Values to PubChemLite

Although quantum CCS prediction methods such as ISiCLE^7^ are generally considered more accurate than ML models, the calculation times are prohibitive for the PubChemLite pipeline. Furthermore, since ISiCLE only predicts values for C, H, N, O, P, and S-containing compounds, CCS values would be missing for ∼40% of PubChemLite. In contrast, the current version of the ML method CCSbase^9^ runs for all but 12 entries (0.003%) of PubChemLite and completes in 3650 s. Calculations are performed and released publicly once a month using the cs3db (https://github.com/dylanhross/c3sdb/)^29^ model (the code behind CCSbase) trained on the data sets listed in Table S1 of the Supporting Information (SI) as published in Ross et al.^9^

Both versions (PubChemLite with and without CCS values) are integrated into MetFrag^22,30^ each month (see SI, Section S2).

### Adding Experimental CCS Values to PubChem

**Figure 2:**
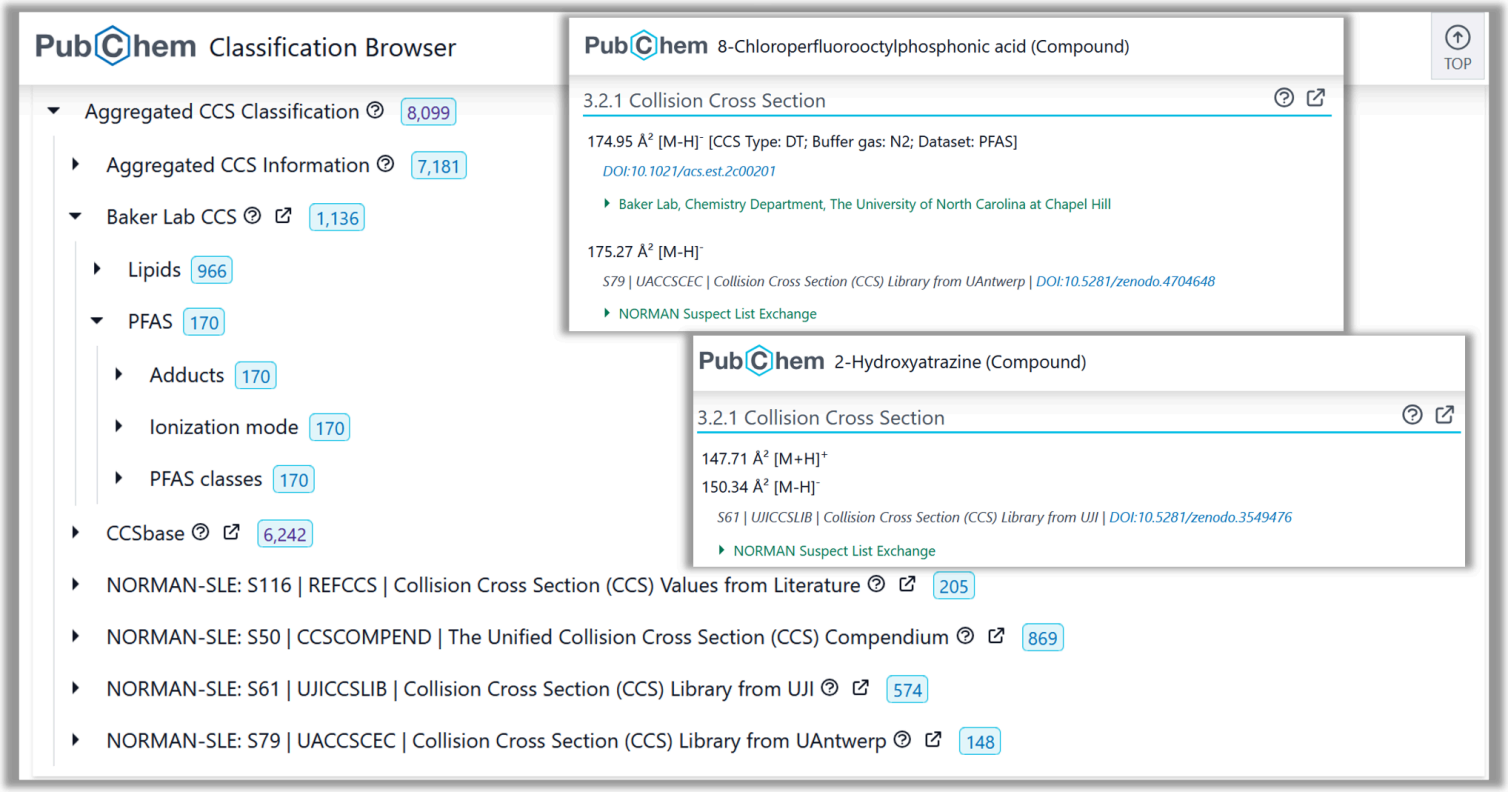
Aggregated Collision Cross Section (CCS) Classification Tree (https://pubchem.ncbi.nlm.nih.gov/classification/#hid=106) in PubChem. Inset: Experimental CCS values in individual PubChem compound records for Cl-PFOPA (CID 138395139) (https://pubchem.ncbi.nlm.nih.gov/compound/138395139#section=Collision-Cross-Section) and the transformation product 2-hydroxyatrazine (CID 135398733) (https://pubchem.ncbi.nlm.nih.gov/compound/135398733#section=Collision-Cross-Section). For high resolution live images, please click the embedded hyperlinks. Logo image from GitLab.^28^

To ensure that ML models have better coverage of environmentally relevant compounds to improve their predictions in the future, part of this work involved establishing a pipeline to integrate experimental CCS values into PubChem. Currently PubChem contains CCS values from the Baker Lab,^31,32^ CCSbase^9^ and four collections via the NORMAN-SLE:^19^ S50 CCSCOMPEND,^33,34^ S61 UJICCSLIB,^35,36^ S79 UACCSCEC,^3,37^ and S116 REFCCS.^38^ These values are displayed on individual records in PubChem and navigable in the PubChem Classification Browser via the CCSbase (https://pubchem.ncbi.nlm.nih.gov/classification/#hid=104),^39^ Baker Lab (https://pubchem.ncbi.nlm.nih.gov/classification/#hid=124),^40^ NORMAN-SLE (https://pubchem.ncbi.nlm.nih.gov/classification/#hid=101)^41^ and the Aggregated CCS (https://pubchem.ncbi.nlm.nih.gov/classification/#hid=106)^42^ trees (see Figure 2). All experimental CCS values are retrieved from PubChem (code available on GitLab (https://gitlab.com/uniluxembourg/lcsb/eci/pubchem/-/blob/master/annotations/CCS/CCS_retrieval)^43^) and archived on Zenodo^44^ after each update.

### PubChemLite Web Interface

**Figure 3:**
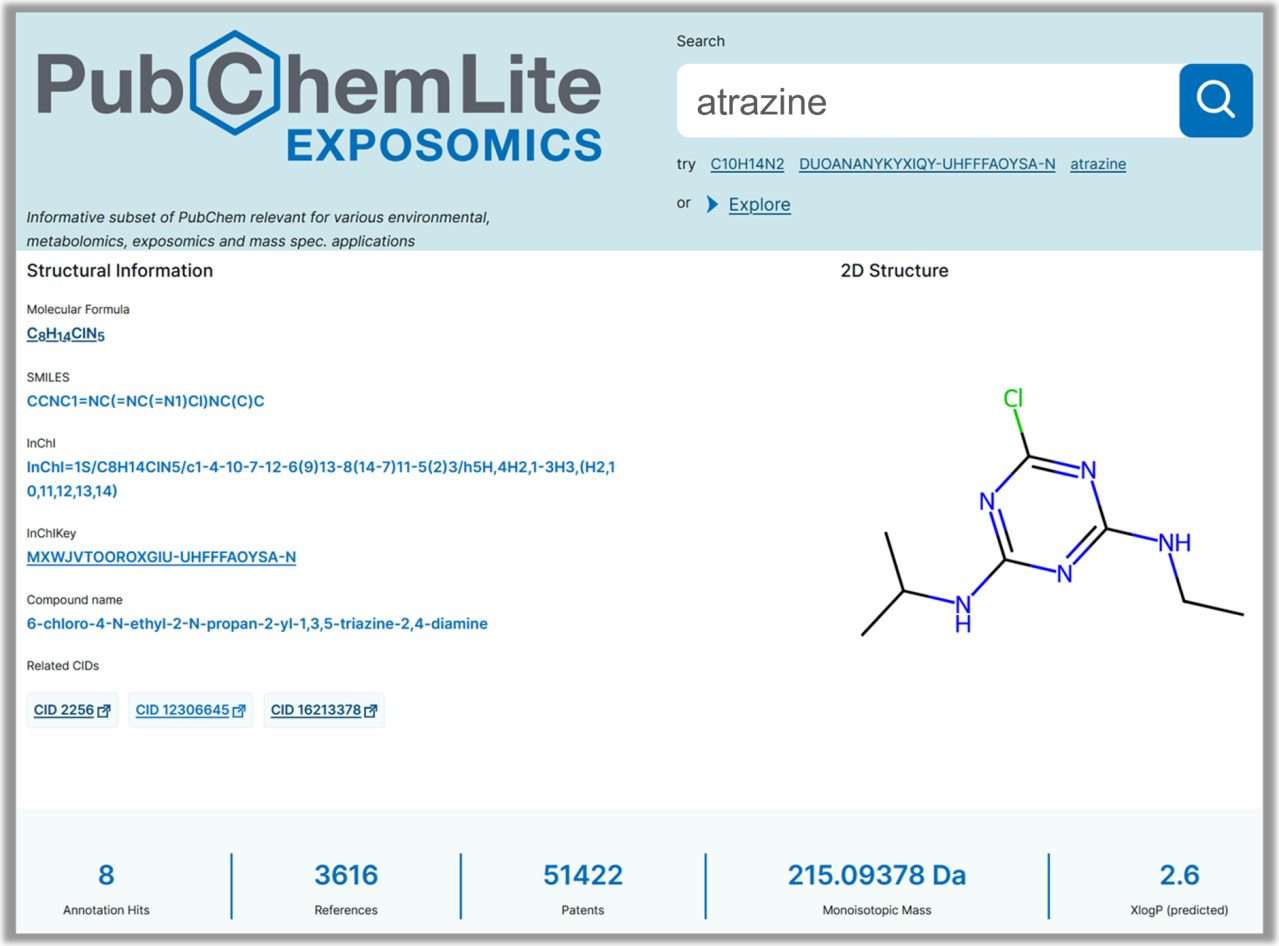
PubChemLite web interface (composite image), compound view of Atrazine (https://pubchemlite.lcsb.uni.lu/e/compound/2256). For high resolution live images, please click the embedded hyperlink. Logo image from GitLab.^28^

**Figure 4:**
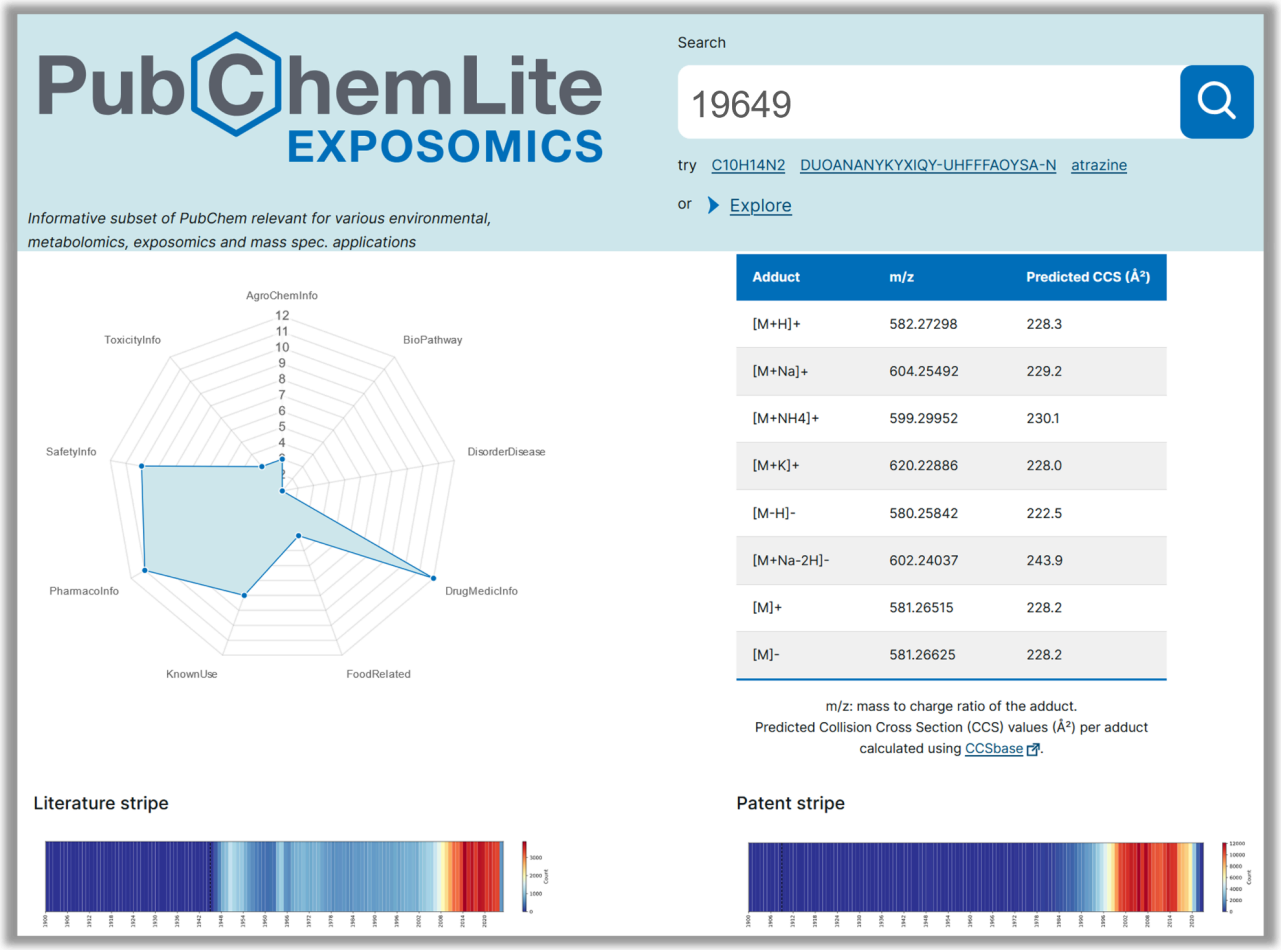
PubChemLite web interface (composite image), view of additional data including annotations, CCS values, and patent and literature stripes for Streptomycin (https://pubchemlite.lcsb.uni.lu/e/compound/19649). For high resolution live images, please click the embedded hyperlink. Logo image from GitLab.^28^

The PubChemLite web interface is developed as a plugin for the ELIXIR-Luxembourg Data Catalog (https://github.com/elixir-luxembourg/data-catalog).^45,46^ It is developed in Python, CSS, HTML and Javascript, using RDKit^47,48^ for structure depiction. For full details, see the PubChemLite-web (https://gitlab.com/uniluxembourg/lcsb/eci/pubchemlite-web) code on GitLab.^49^ The information in the archived PubChemLite-CCSbase CSV files (see Figure 3) is enhanced with additional synonyms from PubChem Downloads^50^ for improved searchability, visualizations of the annotation categories, and tables of the CCS and associated adduct mass values. Finally, historical literature and patent trends are included using the “chemical stripes”^51,52^ where available (see Figure 4). The original R version^53^ was rewritten in Python for integration in PubChemLite-web (https://gitlab.com/uniluxembourg/lcsb/eci/pubchemlite-web),^49^ with code available in both repositories.^49,53^

## Results and Discussion

### PubChemLite Over Time

**Figure 5:**
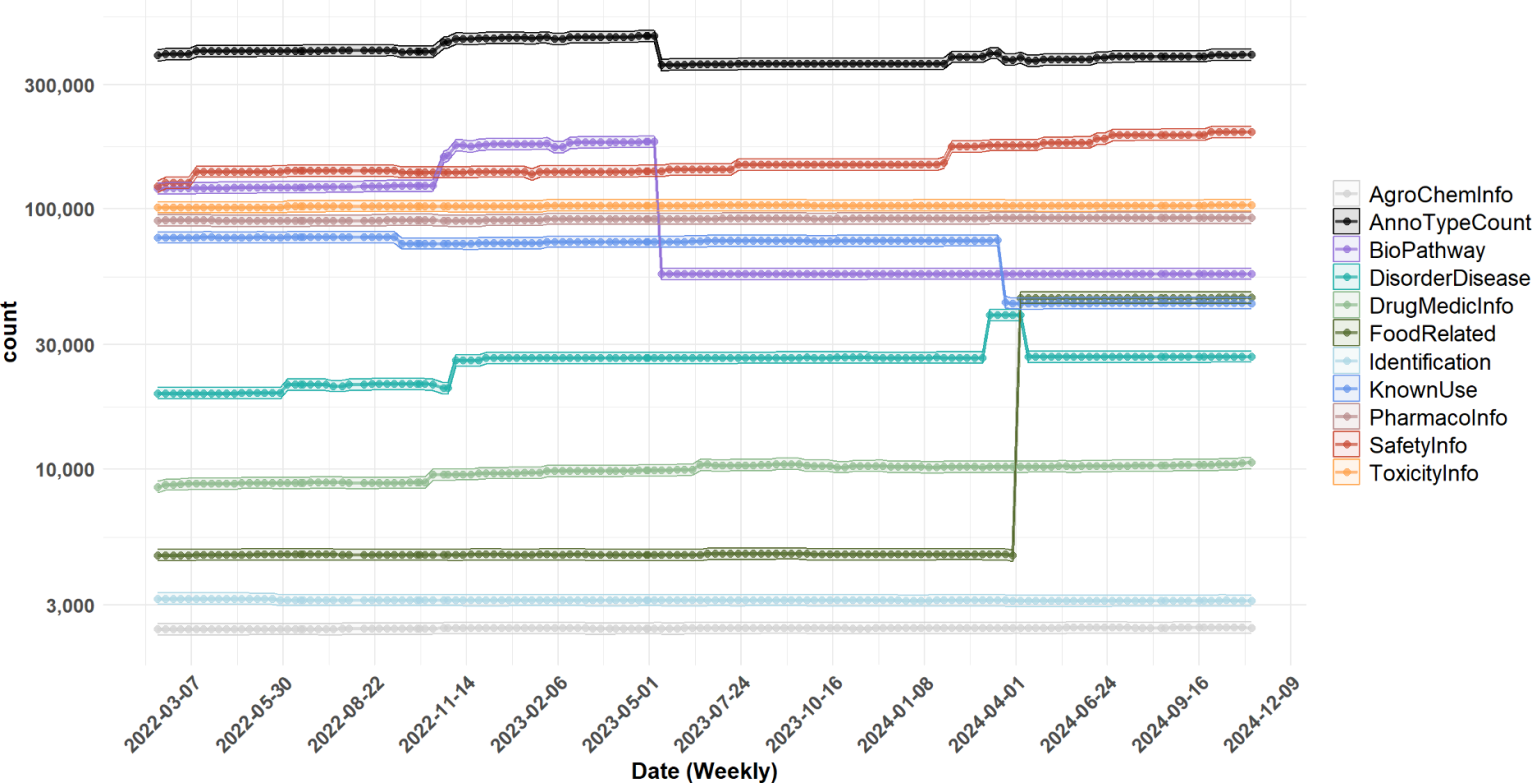
PubChemLite annotation content (total and by category) between 4 Feb. 2022, and 3 Nov. 2024.

The performance of PubChemLite is monitored weekly with every build using the evaluation data set of 977 compounds established in the original publication.^20^ The ranking performance has been quite stable over the three-year period, with median rank = 1 of 794 (81.3%), 1–2 of 917 (93.9%), 1–5 of 960 (98.3%), and 12 (1.2%) failures (compounds absent from PubChemLite due to lack of corresponding annotation). Further details are given in the SI, Section S3, Table S3. The distribution of annotation content included in PubChemLite, including the total number of entries between Feb. 2022 and Nov. 2024, is shown in Figure 5.

Overall, despite PubChem increasing in content dramatically over that time, PubChemLite has remained generally stable at ∼400,000 entries. The systematic increase of the BioPathway category (purple, Figure 5) starting in October 2022 introduced a number of irrelevant candidates in preliminary NTS results^54^ and was alleviated by switching to the “Pathways” subcategory in May 2023 (see Figure 1), improving performance and interpretability of candidate hits. The dramatic increase in FoodRelated information was due to the integration of FooDB^55^ into PubChem annotation content; despite the large increase in that category, the overall candidate numbers remained stable, indicating that many of these candidates already had other annotation content in PubChemLite. The increase and then decrease of content in the DiseaseDisorder category in March–April 2024 was due to an update of one data source that suddenly introduced many low-quality candidates, which was fixed by 12 April (see Figure 5). Overall, the continuous monitoring and use of PubChemLite in various NTS studies helps ensure relevance and usefulness for the community.

### Incorporating CCS Values into Candidate Selection with PubChemLite

The application of PubChemLite with MetFrag using the recommended scoring terms (MetFrag score, MoNA Exact Match, AnnoTypeCount, PubMed_Count, Patent_Count) is described in Section S2 of the SI using an example that highlights how all information could be considered when interpreting candidate results. In this case, the data scores rank one candidate first (carbaryl), whereas experimental data clearly point to desethylterbutylazine as the correct structure. As shown in Table S2, experimental CCS values would distinguish all three candidates (accounting for 1% error), but the predicted values (considering 3% error)^9^ would not (see Section S2, SI for more details). Many cases in the evaluation set are similar, where the predicted values for most candidates are within a 3% prediction error. However, in some cases, some candidates can be eliminated based on predicted CCS values, such as the example of Acemetacin (see Figure S6). These observations match the results of Menger et al. applying PubChemLite-CCS in NTS of mussels.^56^ That study also highlighted discrepancies in predicted CCS values for some environmentally relevant compounds, especially per- and polyfluorinated substances (PFAS). This motivated the collection of the experimental CCS data described in the next paragraph to improve the availability of relevant environmental CCS values for training ML approaches such as the CCSbase.

The experimental CCS data in PubChem currently (5 Nov. 2024) includes a total of 22,192 experimental CCS values corresponding to 8099 unique compounds (CIDs), somewhat smaller than the recently released METLIN-CCS collections.^57,58^ The contributions include 1554 CCS values for 1136 CIDs from the Baker Lab;^31,32^ 17,187 CCS values for 6242 CIDs from CCSbase;^9^ and 3451 CCS values corresponding to 869, 574, 148, and 205 CIDs from the NORMAN-SLE^19^ collections S50 CCSCOMPEND,^33,34^ S61 UJICCSLIB,^35,36^ S79 UACCSCEC,^3,37^ and S116 REFCCS,^38^ respectively. Information is available for 98 adducts, where the most common adducts are [M + H]^+^ (7545 CCS values, 4278 CIDs), [M – H]^−^ (4279 CCS values, 2064 CIDs), [M + Na]^+^ (4064 CCS values, 2831 CIDs), [M + K]^+^ (1179 CCS values, 1140 CIDs), and [M + H – H_2_O]^+^ (1154 CCS values, 1113 CIDs).

### Future Perspectives

PubChemLite continues to develop and improve as a resource for the environmental and exposomics communities based on user feedback. Collaborative research activities have helped trim less relevant content but also identified poor coverage for compounds in sediments, which requires the integration of additional annotation content into PubChem to address fully. Features to be added to PubChemLite in the short term include mass and CCS search options for the web interface and the addition of the “chemical classes” category to include more emerging contaminants like flame retardants. The CCS predictions will be updated once new versions of c3sdb (trained on more data) are available. Separate community efforts are underway to automate identification in NTS using ion mobility data, and the efforts presented here form an important basis for this.

PubChemLite (https://pubchemlite.lcsb.uni.lu) provides efficient candidate sets (tens to hundreds rather than thousands) and information-rich content for interpreting environmental NTS data, with 80% of the evaluation set ranked Top 1 and 98% Top 5, empowering identification in NTS studies. As CCS predictions improve with new technology, in particular, through incorporation of experimental reference values from higher resolving power IMS separations, this performance is likely to improve further. User feedback is welcome (see https://pubchemlite.lcsb.uni.lu/contact).

## Data Availability

The PubChemLite web interface (https://pubchemlite.lcsb.uni.lu) is openly available. PubChemLite is compiled weekly from openly available files downloaded from PubChem^50^ and is archived monthly on Zenodo (DOI: https://doi.org/10.5281/zenodo.5995885). CCS values are added using open cs3db (https://github.com/dylanhross/c3sdb/) code^29^ and the PubChemLite-CCS files are archived on Zenodo at DOI: https://doi.org/10.5281/zenodo.4081056. The Zenodo links redirect to the latest version. The code for the PubChemLite build system (https://gitlab.com/uniluxembourg/lcsb/eci/pclbuild),^25^ inputs (https://gitlab.com/uniluxembourg/lcsb/eci/pubchemlite-input),^24^ chemical stripes (https://gitlab.com/uniluxembourg/lcsb/eci/chemicalstripes)^53^ and interface (https://gitlab.com/uniluxembourg/lcsb/eci/pubchemlite-web)^49^ are openly available on the Environmental Cheminformatics (ECI) GitLab (https://gitlab.com/uniluxembourg/lcsb/eci/).^26^ All resources are available under open licenses, see individual pages for details. This article was submitted as a preprint: Anjana Elapavalore, Dylan Ross, Valentin Groues, Dagny Aurich, Allison Krinsky, Sunghwan Kim, Paul Thiessen, Jian Zhang, James Dodds, Erin Baker, Evan Bolton, Libin Xu, Emma Schymanski. 2024. ChemRxiv. DOI: https://doi.org/10.26434/chemrxiv-2024-2xcsq.

## Supporting Information Available

A document including additional details about the CCSbase training data sets (S1), using PubChemLite in MetFrag (S2), and additional rank and CCS results (S3) (PDF)
