# Supplementary material for: PubChemLite Plus Collision Cross Section (CCS) Values for Enhanced Interpretation of Nontarget Environmental Data
Source: Environ Sci Technol Lett. 2025 Jan 24;12(2):166–74. doi: 10.1021/acs.estlett.4c01003 (PMC11823450; doi:10.1021/acs.estlett.4c01003)
Supplement: Supplementary file 1 — ez4c01003_si_001.pdf [file ez4c01003_si_001.pdf]

Supporting Information for PubChemLite plus Collision Cross Section (CCS) values for enhanced interpretation of non-target environmental data

Anjana Elapavalore[a], Dylan H. Ross[b,c], Valentin Grouès[a], Dagny Aurich[a],
Allison M. Krinsky[b], Sunghwan Kim[d], Paul A. Thiessen[d], Jian Zhang[d], James N. Dodds[e],
Erin S. Baker[e], Evan E. Bolton[d]*, Libin Xu[b]*, Emma L. Schymanski[a]*

[a] Luxembourg Centre for Systems Biomedicine (LCSB), University of Luxembourg, 6 Avenue du Swing, 4367, Belvaux, Luxembourg. ORCIDs AE: 0000-0002-0295-6618; VG: 0000-0001-6501-0806 DA: 0000-0001-8823-0596; ELS: 0000-0001-6868-8145.

[b] Department of Medicinal Chemistry, University of Washington, Seattle, Washington, 98195, United States. ORCIDs DHR: 0009-0005-2943-2282; AMK: NA; LX: 0000-0003-1021-5200.

[c] Current Address: Biological Sciences Division, Pacific Northwest National Laboratory, Richland, 99352, WA, USA.

[d] National Center for Biotechnology Information (NCBI), National Library of Medicine (NLM), National Institutes of Health (NIH), Bethesda, MD, 20894, USA. ORCIDs SK: 0000-0001-9828-2074; PAT: 0000-0002-1992-2086; JZ: 0000-0002-6192-4632; EEB: 0000-0002-5959-6190.

[e] Department of Chemistry, University of North Carolina, Chapel Hill, North Carolina 27599, USA. ORCIDs JND: 0000-0002-9702-2294 ESB: 0000-0001-5246-2213

*Contact author: *Evan E. Bolton*. Street Address: National Center for Biotechnology Information (NCBI), National Library of Medicine (NLM), National Institutes of Health (NIH), Bethesda, MD, 20894, USA. Phone: +1 301 451 1811. Fax: +1 301 480 9241. Email: bolton@ncbi.nlm.nih.gov.

*Contact author: *Libin Xu*. Street Address: Department of Medicinal Chemistry, University of Washington, Seattle, Washington 98195, USA. Phone: +1 206 543-1080. Fax: +1 206 685 3252. Email: libinxu@uw.edu.

*Contact author: *Emma L. Schymanski*. Street Address: Luxembourg Centre for Systems Biomedicine (LCSB), University of Luxembourg, 6 Avenue du Swing, 4367, Belvaux, Luxembourg. Phone: +352 46 66 44 5616. Fax: NA. Email: emma.schymanski@uni.lu

Contents

Section	Description	Pages
S1	CCSbase Traning Datasets	2-4
S2	Using PubChemLite in MetFrag	4-8
S3	Additional Tables and Figures for Results	8-8
	References	9-11

S1 CCSbase Training Datasets

The datasets used to train CCSbase are given in Table S1, named by the internal code used in CCSbase (numbers in superscript are the references; see References). The model was trained as published[1].

Table S1: List of datasets used to train the CCSbase model used in this work.

Ref. Code	Description
zhou1016[2]	Zhou, Z., Shen, X., Tu, J. & Zhu, Z.-J. Large-Scale Prediction of Collision Cross-Section Values for Metabolites in Ion Mobility-Mass Spectrometry. Anal. Chem. 88, 11084-11091 (2016). DOI: 10.1021/acs.analchem.6b03091.
zhou0817[3]	Zhou, Z., Tu, J., Xiong, X., Shen, X. & Zhu, Z.-J. LipidCCS: Prediction of Collision Cross-Section Values for Lipids with High Precision To Support Ion Mobility-Mass Spectrometry-Based Lipidomics. Anal. Chem. 89, 9559-9566 (2017). DOI: 10.1021/acs.analchem.7b02625.
zhen0917[4]	Zheng, X. et al. A structural examination and collision cross section database for over 500 metabolites and xenobiotics using drift tube ion mobility spectrometry. Chem. Sci. 8, 7724-7736 (2017). DOI: 10.1039/C7SC03464D
pagl0314[5]	Paglia, G. et al. Ion Mobility Derived Collision Cross Sections to Support Metabolomics Applications. Anal. Chem. 86, 39850133993 (2014).Paglia, G. et al. Ion Mobility Derived Collision Cross Sections to Support Metabolomics Applications. Anal. Chem. 86, 3985-3993 (2014). DOI: 10.1021/ac500405x
righ0218[6]	Righetti, L. et al. Ion mobility-derived collision cross section database: Application to mycotoxin analysis. Analytica Chimica Acta 1014, 50-57 (2018). DOI: 10.1016/j.aca.2018.01.047
nich1118[7]	Nichols, C. M. et al. Untargeted Molecular Discovery in Primary Metabolism: Collision Cross Section as a Molecular Descriptor in Ion Mobility-Mass Spectrometry. Anal. Chem. 90, 14484-14492 (2018). DOI: 10.1021/acs.analchem.8b04322
may_0114[8]	May, J. C. et al. Conformational Ordering of Biomolecules in the Gas Phase: Nitrogen Collision Cross Sections Measured on a Prototype High Resolution Drift Tube Ion Mobility-Mass Spectrometer. Anal. Chem. 86, 2107-2116 (2014). DOI: 10.1021/ac4038448
moll0218[9]	Mollerup, C. B., Mardal, M., Dalsgaard, P. W., Linnet, K. & Barron, L. P. Prediction of collision cross section and retention time for broad scope screening in gradient reversed-phase liquid chromatography-ion mobility-high resolution accurate mass spectrometry. Journal of Chromatography A 1542, 82-88 (2018). DOI: 10.1016/j.chroma.2018.02.025
hine1217[10]	Hines, K. M. et al. Characterization of the Mechanisms of Daptomycin Resistance among Gram-Positive Bacterial Pathogens by Multidimensional Lipidomics. mSphere 2, 99-16 (2017). DOI: 10.1128/msphere.00492-17
hine0217[11]	Hines, K. M., Herron, J. & Xu, L. Assessment of altered lipid homeostasis by HILIC-ion mobility-mass spectrometry-based lipidomics. The Journal of Lipid Research 58, 809-819 (2017). DOI: 10.1194/jlr.D074724
hine0817[12]	Hines, K. M., Ross, D. H., Davidson, K. L., Bush, M. F. & Xu, L. Large-Scale Structural Characterization of Drug and Drug-Like Compounds by High-Throughput Ion Mobility-

Ref. Code	Description
	Mass Spectrometry. Anal. Chem. 89, 9023-9030 (2017). DOI: 10.1021/acs.analchem.7b01709
groe0815[13]	Groessl, M., Graf, S. & Knochenmuss, R. High resolution ion mobility-mass spectrometry for separation and identification of isomeric lipids. Analyst 140, 6904-6911 (2015). DOI: 10.1039/C5AN00838G
bijl0517[14]	Bijlsma, L. et al. Prediction of Collision Cross-Section Values for Small Molecules: Application to Pesticide Residue Analysis. Anal. Chem. 89, 6583-6589 (2017). DOI: 10.1021/acs.analchem.7b00741
stow0817[15]	Stow, S. M. et al. An Interlaboratory Evaluation of Drift Tube Ion Mobility-Mass Spectrometry Collision Cross Section Measurements. Anal. Chem. 89, 9048-9055 (2017). DOI: 10.1021/acs.analchem.7b01729
hine0119[16]	Hines, K. M. & Xu, L. Lipidomic consequences of phospholipid synthesis defects in Escherichia coli revealed by HILIC-ion mobility-mass spectrometry. Chemistry and Physics of Lipids 219, 15-22 (2019). DOI: 10.1016/j.chemphyslip.2019.01.007
leap0219[17]	Leaptrot, K. L., May, J. C., Dodds, J. N. & McLean, J. A. Ion mobility conformational lipid atlas for high confidence lipidomics. Nature Communications 1-9 (2019). DOI: 10.1038/s41467-019-08897-5
blaz0818[18]	Blazeenovic, I. et al. Increasing Compound Identification Rates in Untargeted Lipidomics Research with Liquid Chromatography Drift Time-Ion Mobility Mass Spectrometry. Anal. Chem. 90, 10758-10764 (2018). DOI: 10.1021/acs.analchem.8b01527
vasi0120[19]	Vasilopoulou, C. G. et al. Trapped ion mobility spectrometry and PASEF enable in-depth lipidomics from minimal sample amounts. Nature Communications 1-11 (2020). DOI: 10.1038/s41467-019-14044-x
tsug0220[20,21]	Tsugawa, H. et al. MS-DIAL 4: accelerating lipidomics using an MS/MS, CCS, and retention time atlas. bioRxiv 37, 513 (2020). DOI: 10.1101/2020.02.11.944900
lian0118[22]	Lian, R. et al. Ion mobility derived collision cross section as an additional measure to support the rapid analysis of abused drugs and toxic compounds using electrospray ion mobility time-of-flight mass spectrometry. Anal. Methods 10, 749-756 (2018). DOI: 10.1039/C7AY02808C
teja0918[23]	Tejada-Casado, C. et al. Collision cross section (CCS) as a complementary parameter to characterize human and veterinary drugs. Analytica Chimica Acta 1043, 52-63 (2018). DOI: 10.1016/j.aca.2018.09.065
pola0620[24]	Poland, J. C. et al. Collision Cross Section Conformational Analyses of Bile Acids via Ion Mobility-Mass Spectrometry. Journal of the American Society for Mass Spectrometry 31, 1625-1631 (2020). DOI: 10.1021/jasms.0c00015
dodd0220[25]	Dodds, J. et al. Rapid Characterization of Per- and Polyfluoroalkyl Substances (PFAS) by Ion Mobility Spectrometry-Mass Spectrometry (IMS-MS). Anal. Chem. 92, 4427-4435 (2020). DOI: 10.1021/acs.analchem.9b05364
celm1120[26]	Celma, A. et al. Improving Target and Suspect Screening High-Resolution Mass Spectrometry Workflows in Environmental Analysis by Ion Mobility Separation. Environ. Sci. Technol. 54, 15120-15131 (2020). DOI: 10.1021/acs.est.0c05713
belo0321[27]	Belova, L. et al. Ion Mobility-High-Resolution Mass Spectrometry (IM-HRMS) for the

Ref. Code	Description
	Analysis of Contaminants of Emerging Concern (CECs): Database Compilation and Application to Urine Samples. Anal. Chem. 93, 16, 6428-6436 (2021). DOI: 10.1021/acs.analchem.1c00142
ross0422[28]	Dylan H. Ross, Ryan P. Seguin, Allison M. Krinsky, and Libin Xu Journal of the American Society for Mass Spectrometry. 33, 6, 1061-1072 (2022). DOI: 10.1021/jasms.2c00111

S2 Using PubChemLite in MetFrag

The latest versions of PubChemLite and PubChemLite-CCSbase are integrated into the MetFrag web interface each month via pull request. These are then available to chose from the dropdown menu under the “Local Databases” option (typically appearing at the top of the list). See Figure S1.

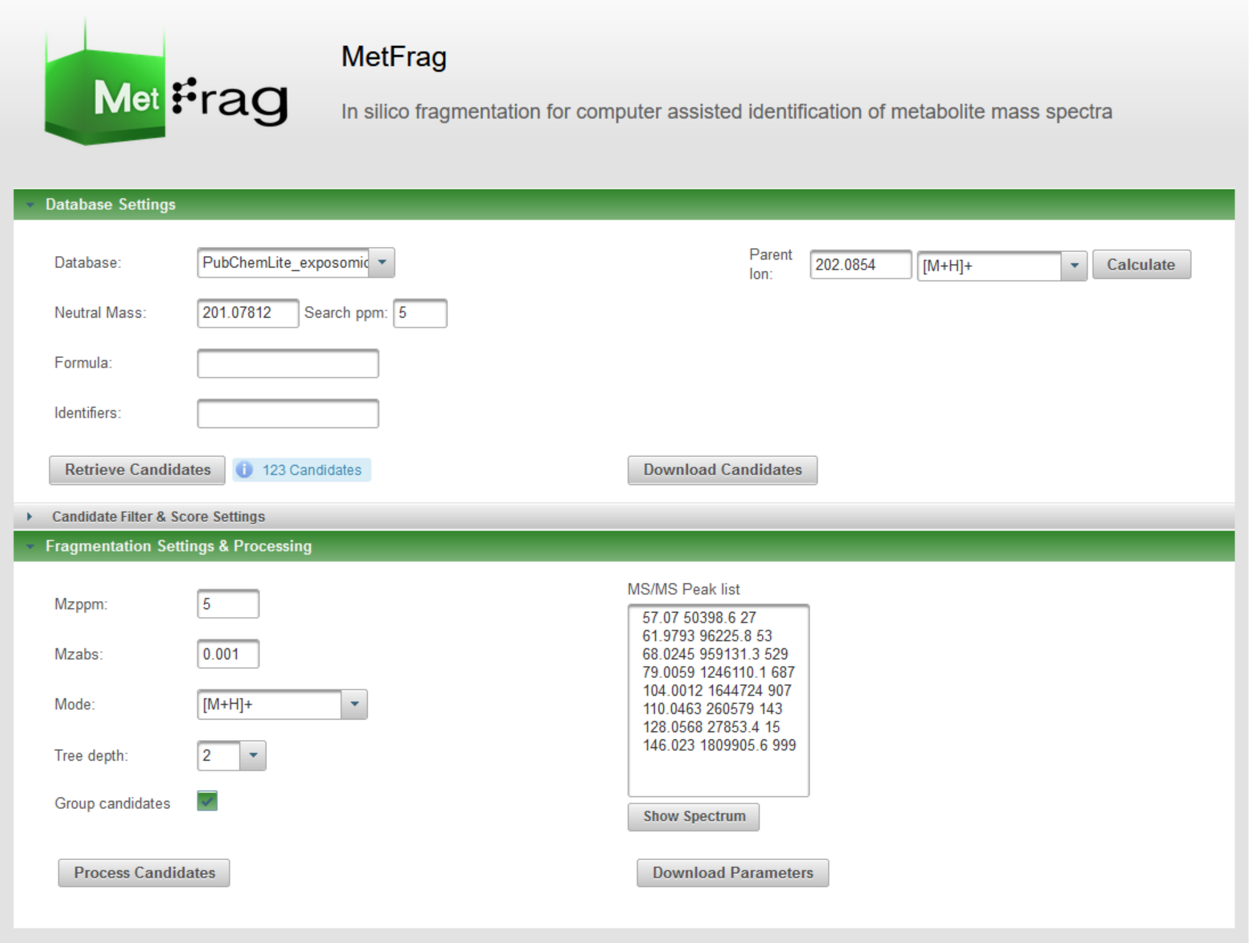

Figure S1: Querying PubChemLite in MetFrag with desethylterbutylazine, mass spectrum MSBNK-Eawag-EA067107.

Once candidates are retrieved, it is possible to expand the “Candidate Filter & Score Settings” bar (see Figure S2). Select the “Exact Spectral Similarity (MoNA)” option to add an exact spectral match to the scoring terms (recommended to achieve a Level 2a Spectral Match confidence level[29] with a sufficiently high score). Generally an Exact MoNA score >0.9 indicates an extremely good match, although values >0.8 can also indicate a good match if the spectra are recorded on different instruments.

When using PubChemLite, three additional scoring terms are highly recommended to achieve the evaluation performance mentioned in the main article. These are the “AnnoTypeCount”, “Patent_Count” and “PubMed_Count” terms listed under the “Database Scoring Terms” menu (see Figure S2, the selected categories are marked in green).

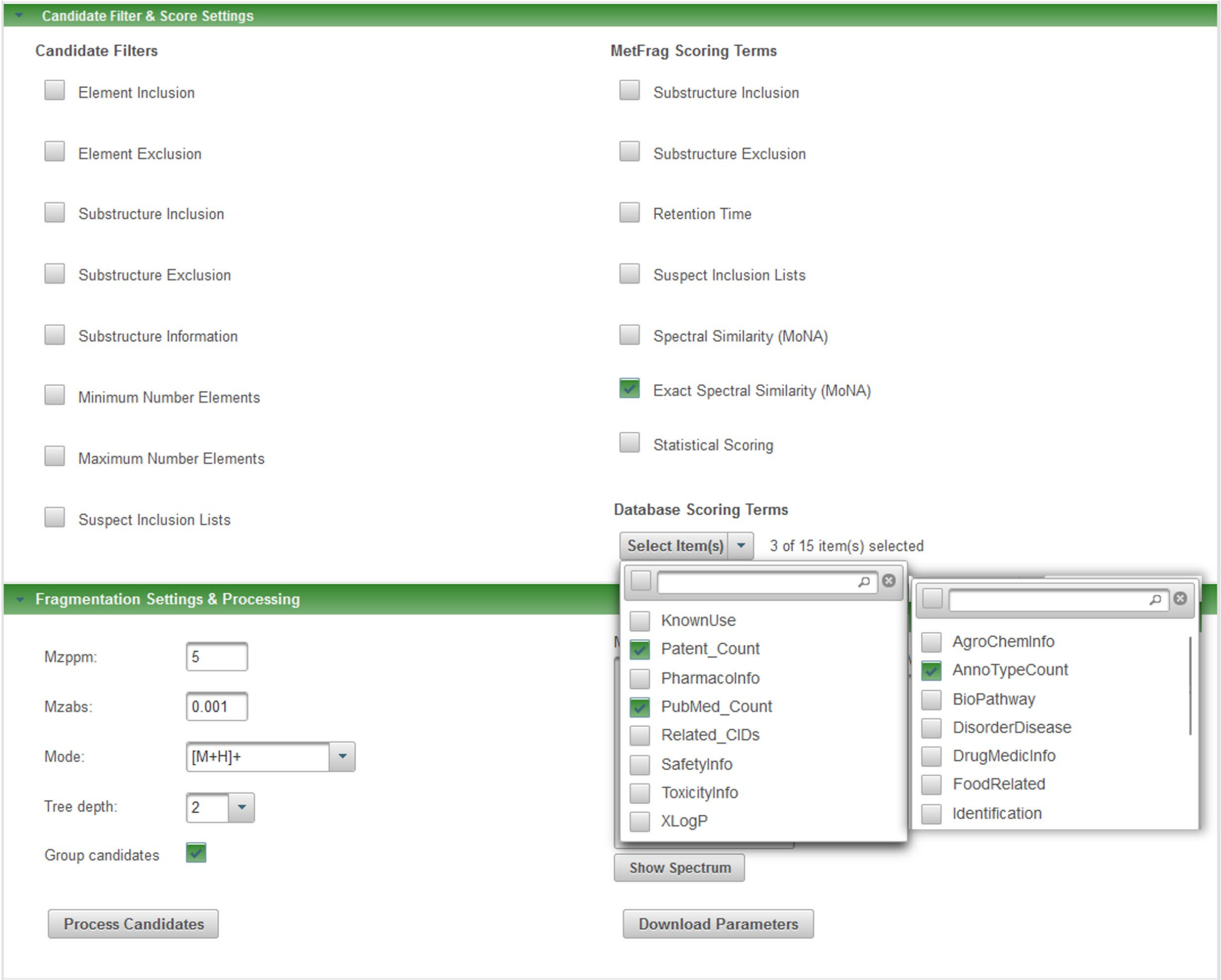

Figure S2: Recommended Candidate Filter & Scoring Settings for PubChemLite in MetFrag. Selected entries are indicated with the green box.

The other “Database Scoring Terms” indicate the specific annotation categories and can be used to selectively up-weight entries that are e.g. agrochemicals (“AgroChemInfo”) or pharmaceuticals (“DrugMedicInfo”), but may not maintain the same ranking performance.

Once candidates are processed, the “Statistics” tab can be used to get an overall impression of the results via the “Candidate Score Distribution” plot (see Figure S3). The candidates can be viewed in the “Results” tab. Figure S4 shows the candidates with all scoring terms equally weighted, while Figure S5 shows the candidates considering only the experimental terms (the MetFrag and MoNA scores). In this case, the experimental evidence clearly supports the candidate desethylterbutylazine, despite other candidates with higher patent and literature scores.

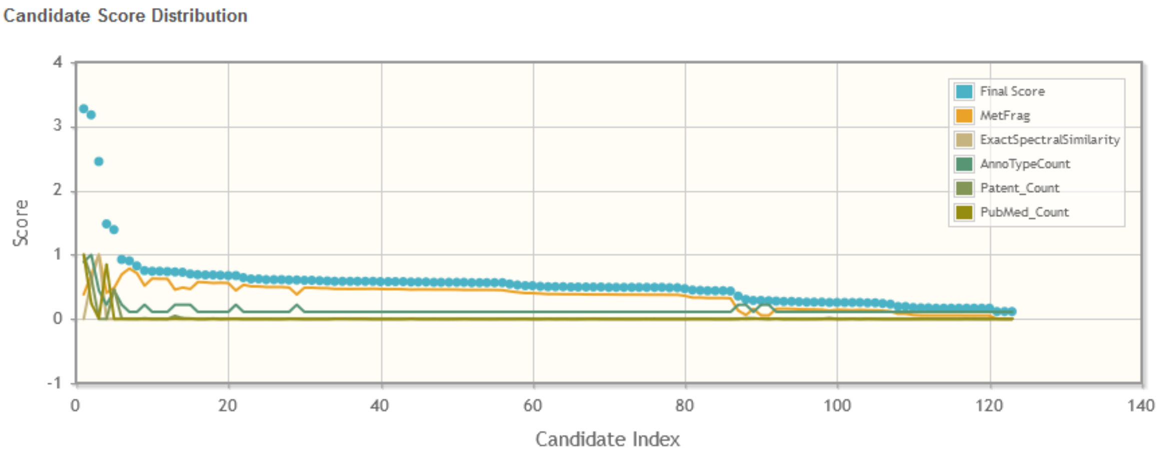

Figure S3: Candidate Score Distribution for the example of desethylterbutylazine with PubChemLite in MetFrag.

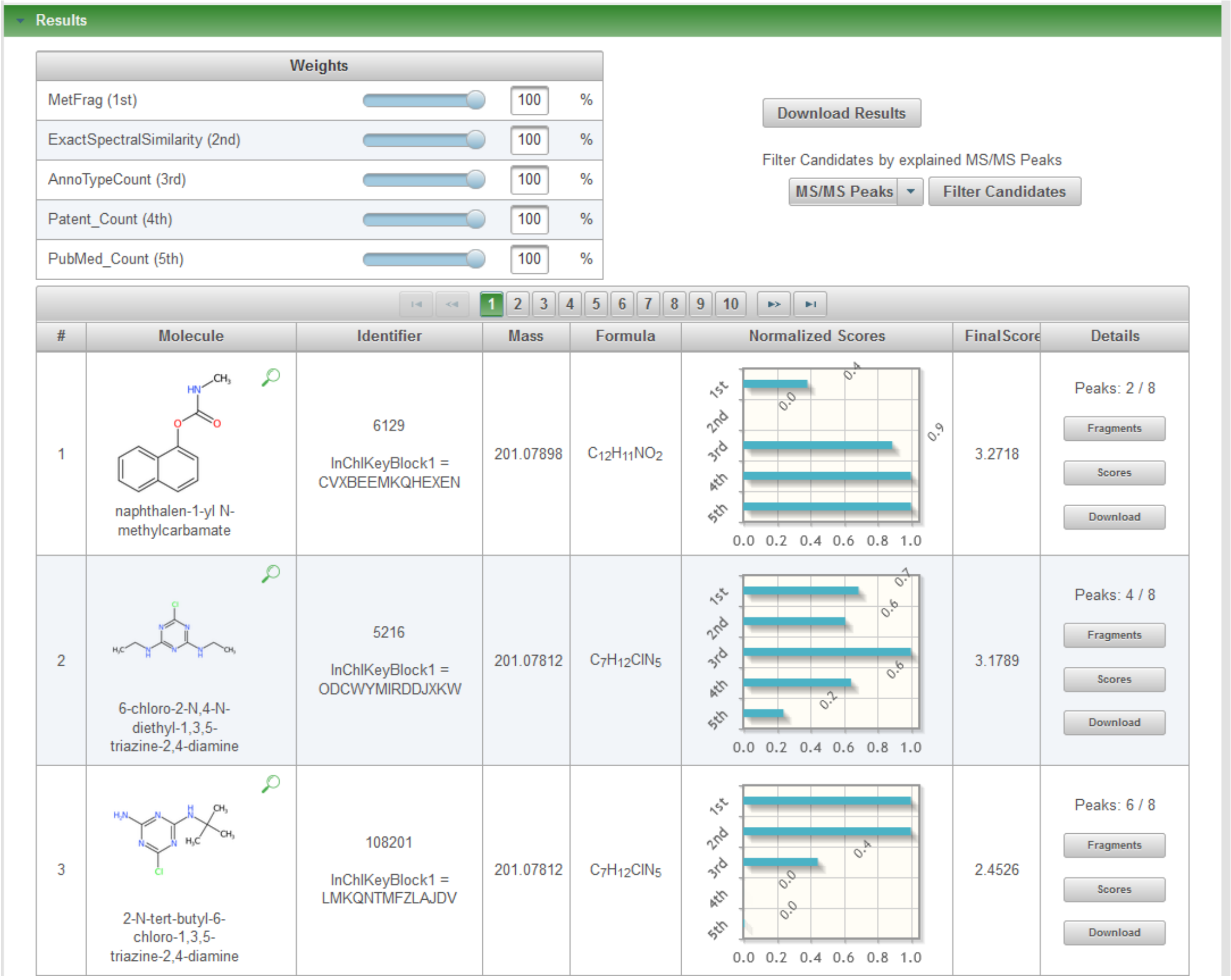

Figure S4: Results tab with default scores for the example of desethylterbutylazine with PubChemLite in MetFrag. Corresponding CCS values are shown in Table S2.

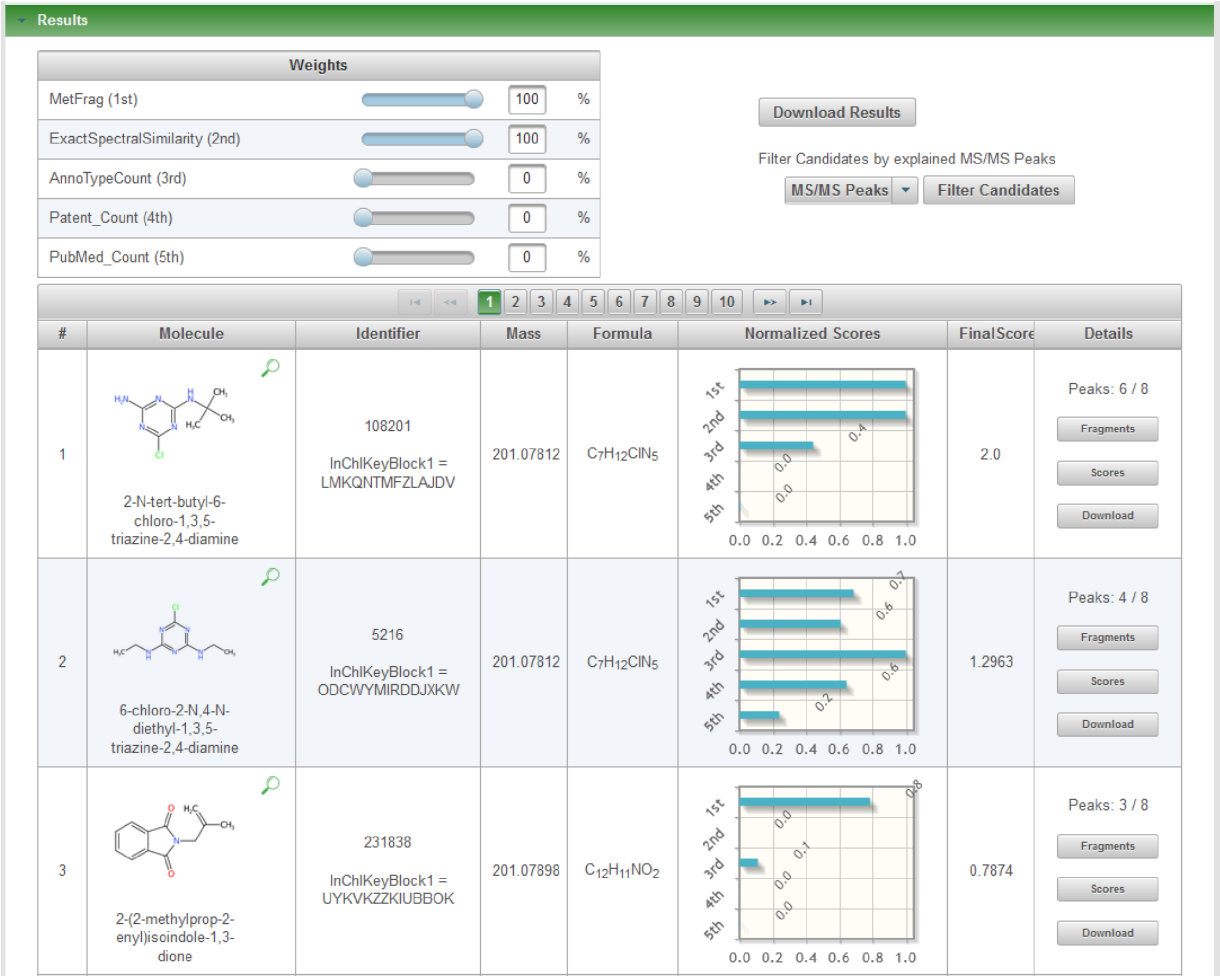

Figure S5: Results tab with experimental scores only for the example of desethylterbutylazine with PubChemLite in MetFrag.

Table S2 shows an overview of the experimental and predicted collision cross section (CCS) values for the top three candidates in Figure S4. This shows that while each candidate could be distinguished with experimental CCS (accounting for 1% error), the predicted error is too large to reliably distinguish simazine from desethylterbutylazine, although the relative order is the same as the experimental values.

Table S2: Overview of Collision Cross Section (CCS) data for the three top candidates in Figure S4. Ranges calculated using 1% error for experimental and 3% error for predicted values.

Name	Desethylterbutylazine	Simazine	Carbaryl
CID	108201	5216	6129
m/z $[M+H]^+$	202.0854	202.0854	202.0863
Experimental CCS[26]	144.71	143	147.98
CCS Range (experimental)	[143.26; 146.16]	[141.57; 144.43]	[146.50; 149.46]
Predicted CCS	142.5	141.2	142.1
CCS Range (predicted)	[138.22; 146.77]	[136.96; 145.44]	[137.83; 146.36]

If starting from the experimental CCS value of carbaryl, both simazine and desethylterbutylazine would be excluded based on the predictions, but unfortunately so would carbaryl. This is in agreement with observations made in earlier studies[30] (especially notable for per- and polyfluorinated substances) and indicates the need for larger experimental datasets to improve predictions further, as compiled in this study (and others).

S3 Additional Tables and Figures for Results

PubChemLite Over Time

Additional details about the ranking performance of PubChemLite over time are given in Table S3. The performance of October 2020 was the time point of the original publication[31], while October 2024 corresponds to the current version at the time of original submission. The current performance is close to the median values and slightly better than the original publication.

Table S3: Performance of PubChemLite over time for the original evaluation dataset[31].

	Oct. 2020	Oct. 2024	Minimum	Maximum	Median	Median (%)
Rank 1	794	797	788	800	794	81.3%
Rank 1-2	912	916	909	922	917	93.9%
Rank 1-5	954	960	955	963	960	98.3%
Failures	15	11	10	15	12	1.2%
Total	977	977	977	977	977	100%

Figure S6 shows the example of Acemetacin, one case where the predicted CCS values help eliminate the second-ranked candidate from contention during identification with PubChemLite and MetFrag.

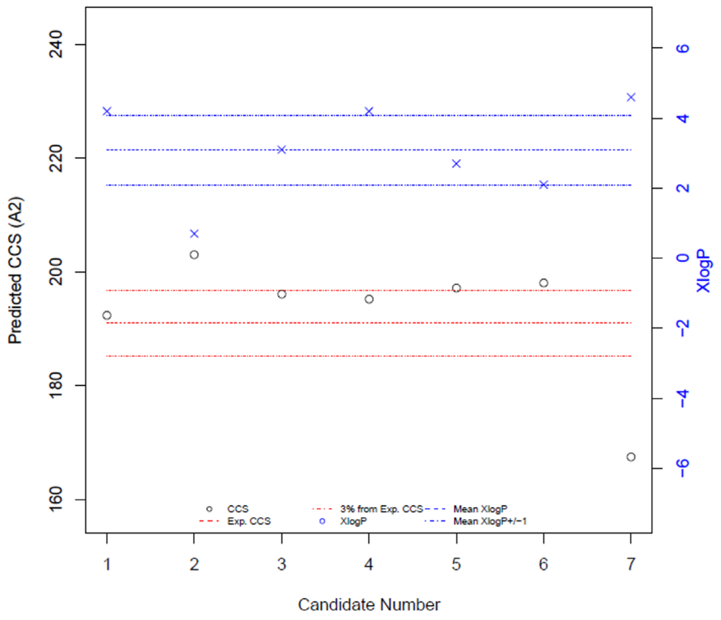

Figure S6: Eliminating candidates based on CCS and XlogP values with the example of Acemetacin[32].

References

(1) Ross, D. H.; Cho, J. H.; Xu, L. Breaking Down Structural Diversity for Comprehensive Prediction of Ion-Neutral Collision Cross Sections. *Analytical Chemistry* **2020**, *92* (6), 4548–4557. https://doi.org/10.1021/acs.analchem.9b05772.

(2) Zhou, Z.; Shen, X.; Tu, J.; Zhu, Z.-J. Large-Scale Prediction of Collision Cross-Section Values for Metabolites in Ion Mobility-Mass Spectrometry. *Analytical Chemistry* **2016**, *88* (22), 11084–11091. https://doi.org/10.1021/acs.analchem.6b03091.

(3) Zhou, Z.; Tu, J.; Xiong, X.; Shen, X.; Zhu, Z.-J. LipidCCS: Prediction of Collision Cross-Section Values for Lipids with High Precision To Support Ion Mobility–Mass Spectrometry-Based Lipidomics. *Analytical Chemistry* **2017**, *89* (17), 9559–9566. https://doi.org/10.1021/acs.analchem.7b02625.

(4) Zheng, X.; Aly, N. A.; Zhou, Y.; Dupuis, K. T.; Bilbao, A.; Paurus, V. L.; Orton, D. J.; Wilson, R.; Payne, S. H.; Smith, R. D.; Baker, E. S. A Structural Examination and Collision Cross Section Database for over 500 Metabolites and Xenobiotics Using Drift Tube Ion Mobility Spectrometry. *Chem. Sci.* **2017**, *8* (11), 7724–7736. https://doi.org/10.1039/C7SC03464D.

(5) Paglia, G.; Williams, J. P.; Menikarachchi, L.; Thompson, J. W.; Tyldesley-Worster, R.; Halldórsson, S.; Rolfsson, O.; Moseley, A.; Grant, D.; Langridge, J.; Palsson, B. O.; Astarita, G. Ion Mobility Derived Collision Cross Sections to Support Metabolomics Applications. *Analytical Chemistry* **2014**, *86* (8), 3985–3993. https://doi.org/10.1021/ac500405x.

(6) Righetti, L.; Bergmann, A.; Galaverna, G.; Rolfsson, O.; Paglia, G.; Dall'Asta, C. Ion Mobility-Derived Collision Cross Section Database: Application to Mycotoxin Analysis. *Analytica Chimica Acta* **2018**, *1014*, 50–57. https://doi.org/10.1016/j.aca.2018.01.047.

(7) Nichols, C. M.; Dodds, J. N.; Rose, B. S.; Picache, J. A.; Morris, C. B.; Codreanu, S. G.; May, J. C.; Sherrod, S. D.; McLean, J. A. Untargeted Molecular Discovery in Primary Metabolism: Collision Cross Section as a Molecular Descriptor in Ion Mobility-Mass Spectrometry. *Analytical Chemistry* **2018**, *90* (24), 14484–14492. https://doi.org/10.1021/acs.analchem.8b04322.

(8) May, J. C.; Goodwin, C. R.; Lareau, N. M.; Leaptrot, K. L.; Morris, C. B.; Kurulugama, R. T.; Mordehai, A.; Klein, C.; Barry, W.; Darland, E.; Overney, G.; Imatani, K.; Stafford, G. C.; Fjeldsted, J. C.; McLean, J. A. Conformational Ordering of Biomolecules in the Gas Phase: Nitrogen Collision Cross Sections Measured on a Prototype High Resolution Drift Tube Ion Mobility-Mass Spectrometer. *Analytical Chemistry* **2014**, *86* (4), 2107–2116. https://doi.org/10.1021/ac4038448.

(9) Mollerup, C. B.; Mardal, M.; Dalsgaard, P. W.; Linnet, K.; Barron, L. P. Prediction of Collision Cross Section and Retention Time for Broad Scope Screening in Gradient Reversed-Phase Liquid Chromatography-Ion Mobility-High Resolution Accurate Mass Spectrometry. *Journal of Chromatography A* **2018**, *1542*, 82–88. https://doi.org/10.1016/j.chroma.2018.02.025.

(10) Hines, K. M.; Waalkes, A.; Penewit, K.; Holmes, E. A.; Salipante, S. J.; Werth, B. J.; Xu, L. Characterization of the Mechanisms of Daptomycin Resistance Among Gram-Positive Bacterial Pathogens by Multidimensional Lipidomics. *mSphere* **2017**, *2* (6), e00492–17. https://doi.org/10.1128/mSphere.00492-17.

(11) Hines, K. M.; Herron, J.; Xu, L. Assessment of Altered Lipid Homeostasis by HILIC-Ion Mobility-Mass Spectrometry-Based Lipidomics. *Journal of Lipid Research* **2017**, *58* (4), 809–819. https://doi.org/10.1194/jlr.D074724.

(12) Hines, K. M.; Ross, D. H.; Davidson, K. L.; Bush, M. F.; Xu, L. Large-Scale Structural Characterization of Drug and Drug-Like Compounds by High-Throughput Ion Mobility-Mass Spectrometry. *Analytical Chemistry* **2017**, *89* (17), 9023–9030. https://doi.org/10.1021/acs.analchem.7b01709.

(13) Groessl, M.; Graf, S.; Knochenmuss, R. High Resolution Ion Mobility-Mass Spectrometry for Separation and Identification of Isomeric Lipids. *The Analyst* **2015**, *140* (20), 6904–6911. https://doi.org/10.1039/C5AN00838G.

(14) Bijlsma, L.; Bade, R.; Celma, A.; Mullin, L.; Cleland, G.; Stead, S.; Hernandez, F.; Sancho, J. V. Prediction of Collision Cross-Section Values for Small Molecules: Application to Pesticide Residue Analysis. *Analytical Chemistry* **2017**, *89* (12), 6583–6589. https://doi.org/10.1021/acs.analchem.7b00741.

(15) Stow, S. M.; Causon, T. J.; Zheng, X.; Kurulugama, R. T.; Mairinger, T.; May, J. C.; Rennie, E. E.; Baker, E. S.; Smith, R. D.; McLean, J. A.; Hann, S.; Fjeldsted, J. C. An Interlaboratory Evaluation of Drift Tube Ion Mobility–Mass Spectrometry Collision Cross Section Measurements. *Analytical Chemistry* **2017**, *89* (17), 9048–9055. https://doi.org/10.1021/acs.analchem.7b01729.

(16) Hines, K. M.; Xu, L. Lipidomic Consequences of Phospholipid Synthesis Defects in Escherichia Coli Revealed by HILIC-Ion Mobility-Mass Spectrometry. *Chemistry and Physics of Lipids* **2019**, *219*, 15–22. https://doi.org/10.1016/j.chemphyslip.2019.01.007.

(17) Leaptrot, K. L.; May, J. C.; Dodds, J. N.; McLean, J. A. Ion Mobility Conformational Lipid Atlas for High Confidence Lipidomics. *Nature Communications* **2019**, *10* (1), 985. https://doi.org/10.1038/s41467-019-08897-5.

(18) Blaženović, I.; Shen, T.; Mehta, S. S.; Kind, T.; Ji, J.; Piparo, M.; Cacciola, F.; Mondello, L.; Fiehn, O. Increasing Compound Identification Rates in Untargeted Lipidomics Research with Liquid Chromatography Drift Time–Ion Mobility Mass Spectrometry. *Analytical Chemistry* **2018**, *90* (18), 10758–10764. https://doi.org/10.1021/acs.analchem.8b01527.

(19) Vasilopoulou, C. G.; Sulek, K.; Brunner, A.-D.; Meitei, N. S.; Schweiger-Hufnagel, U.; Meyer, S. W.; Barsch, A.; Mann, M.; Meier, F. Trapped Ion Mobility Spectrometry and PASEF Enable in-Depth Lipidomics from Minimal Sample Amounts. *Nature Communications* **2020**, *11* (1), 331. https://doi.org/10.1038/s41467-019-14044-x.

(20) Tsugawa, H.; Ikeda, K.; Takahashi, M.; Satoh, A.; Mori, Y.; Uchino, H.; Okahashi, N.; Yamada, Y.; Tada, I.; Bonini, P.; Higashi, Y.; Okazaki, Y.; Zhou, Z.; Zhu, Z.-J.; Koelmel, J.; Cajka, T.; Fiehn, O.; Saito, K.; Arita, M.; Arita, M. A Lipidome Atlas in MS-DIAL 4. *Nature Biotechnology* **2020**, *38* (10), 1159–1163. https://doi.org/10.1038/s41587-020-0531-2.

(21) Tsugawa, H.; Ikeda, K.; Takahashi, M.; Satoh, A.; Mori, Y.; Uchino, H.; Okahashi, N.; Yamada, Y.; Tada, I.; Bonini, P.; Higashi, Y.; Okazaki, Y.; Zhou, Z.; Zhu, Z.-J.; Koelmel, J.; Cajka, T.; Fiehn, O.; Saito, K.; Arita, M.; Arita, M. MS-DIAL 4: Accelerating Lipidomics Using an MS/MS, CCS, and Retention Time Atlas, 2020. https://doi.org/10.1101/2020.02.11.944900.

(22) Lian, R.; Zhang, F.; Zhang, Y.; Wu, Z.; Ye, H.; Ni, C.; Lv, X.; Guo, Y. Ion Mobility Derived Collision Cross Section as an Additional Measure to Support the Rapid Analysis of Abused Drugs and Toxic Compounds Using Electrospray Ion Mobility Time-of-Flight Mass Spectrometry. *Analytical Methods* **2018**, *10* (7), 749–756. https://doi.org/10.1039/C7AY02808C.

(23) Tejada-Casado, C.; Hernández-Mesa, M.; Monteau, F.; Lara, F. J.; Olmo-Iruela, M. D.; García-Campaña, A. M.; Le Bizec, B.; Dervilly-Pinel, G. Collision Cross Section (CCS) as a Complementary Parameter to Characterize Human and Veterinary Drugs. *Analytica Chimica Acta* **2018**, *1043*, 52–63. https://doi.org/10.1016/j.aca.2018.09.065.

(24) Poland, J. C.; Leaptrot, K. L.; Sherrod, S. D.; Flynn, C. R.; McLean, J. A. Collision Cross Section Conformational Analyses of Bile Acids via Ion Mobility–Mass Spectrometry. *Journal of the American Society for Mass Spectrometry* **2020**, *31* (8), 1625–1631. https://doi.org/10.1021/jasms.0c00015.

(25) Dodds, J. N.; Hopkins, Z. R.; Knappe, D. R. U.; Baker, E. S. Rapid Characterization of Per- and Polyfluoroalkyl Substances (PFAS) by Ion Mobility Spectrometry–Mass Spectrometry (IMS-MS). *Analytical Chemistry* **2020**, *92* (6), 4427–4435. https://doi.org/10.1021/acs.analchem.9b05364.

(26) Celma, A.; Sancho, J. V.; Schymanski, E. L.; Fabregat-Safont, D.; Ibáñez, M.; Goshawk, J.; Barknowitz, G.; Hernández, F.; Bijlsma, L. Improving Target and Suspect Screening High-Resolution Mass Spectrometry Workflows in Environmental Analysis by Ion Mobility Separation. *Environmental Science & Technology* **2020**, *54* (23), 15120–15131. https://doi.org/10.1021/acs.est.0c05713.

(27) Belova, L.; Caballero-Casero, N.; Nuijs, A. L. N. van; Covaci, A. Ion Mobility-High-Resolution Mass Spectrometry (IM-HRMS) for the Analysis of Contaminants of Emerging Concern (CECs): Database Compilation and Application to Urine Samples. *Analytical Chemistry* **2021**, *93* (16), 6428–6436. https://doi.org/10.1021/acs.analchem.1c00142.

(28) Ross, D. H.; Seguin, R. P.; Krinsky, A. M.; Xu, L. High-Throughput Measurement and Machine Learning-Based Prediction of Collision Cross Sections for Drugs and Drug Metabolites. *Journal of the American Society for Mass Spectrometry* **2022**, *33* (6), 1061–1072. https://doi.org/10.1021/jasms.2c00111.

(29) Schymanski, E. L.; Jeon, J.; Gulde, R.; Fenner, K.; Ruff, M.; Singer, H. P.; Hollender, J. Identifying Small Molecules via High Resolution Mass Spectrometry: Communicating Confidence. *Environmental Science & Technology* **2014**, *48* (4), 2097–2098. https://doi.org/10.1021/es5002105.

(30) Menger, F.; Celma, A.; Schymanski, E. L.; Lai, F. Y.; Bijlsma, L.; Wiberg, K.; Hernández, F.; Sancho, J. V.; Ahrens, L. Enhancing Spectral Quality in Complex Environmental Matrices: Supporting Suspect and Non-Target Screening in Zebra Mussels with Ion Mobility. *Environment International* **2022**, *170*, 107585. https://doi.org/10.1016/j.envint.2022.107585.

(31) Schymanski, E. L.; Kondić, T.; Neumann, S.; Thiessen, P. A.; Zhang, J.; Bolton, E. E. Empowering Large Chemical Knowledge Bases for Exposomics: PubChemLite Meets MetFrag. *Journal of Cheminformatics* **2021**, *13* (1), 19. https://doi.org/10.1186/s13321-021-00489-0.

(32) Schymanski, E. SMS Keynote: Navigating Millions of Chemicals in Metabolomics and Exposomics Workflows. *Zenodo* **2023**. https://doi.org/10.5281/zenodo.8343923.
